# How monkeys carve up the visual world

**DOI:** 10.7554/eLife.112456

**Published:** 2026-08-17

**Authors:** Binxu Wang

**Affiliations:** 1 Kempner Institute, Harvard University Allston United States

**Keywords:** vision, categories, neural networks, generalisation, Rhesus macaque

## Abstract

Monkeys generalize many visual categorization rules, such as animate versus inanimate, but fail on culturally defined ones, placing their behavior closer to networks trained on images alone than to humans.

**Related research article** Zhang H, Zheng Z, Hu J, Wang Q, Xu M, Zhou Z, Li Z, Okazawa G. 2026. A battery of image classification challenges reveals shared and distinct object categorization behavior across monkeys, humans, and deep networks. *eLife* **15**:RP111725. doi: 10.7554/eLife.111725.

The visual world is a continuum. To act within it, we must divide what we see into useful categories. For example, a driver who sees something on the road must quickly decide if it is a pedestrian, animal or harmless debris. Humans and monkeys make such judgments after a glance, and artificial neural networks now classify images with comparable accuracy (Rajalingham et al., 2018).

However, generalization – how the learner treats cases it has never seen – reveals what has actually been learned and how they have carved up the visual world. For example, when they see a flamingo in one group and a tuna in the other, the intended rule separating them may be "bird versus fish" (Figure 1A). Yet the same examples equally support "pink versus grey", or "flies versus swims". If then presented with a penguin, a learner that inferred "bird versus fish" groups it with the flamingo, while one that inferred "flies versus swims" groups it with the tuna. Both rules fit the training examples perfectly, and only the new case tells us which one the learner infers (Goodman, 1955).

**Figure 1.**
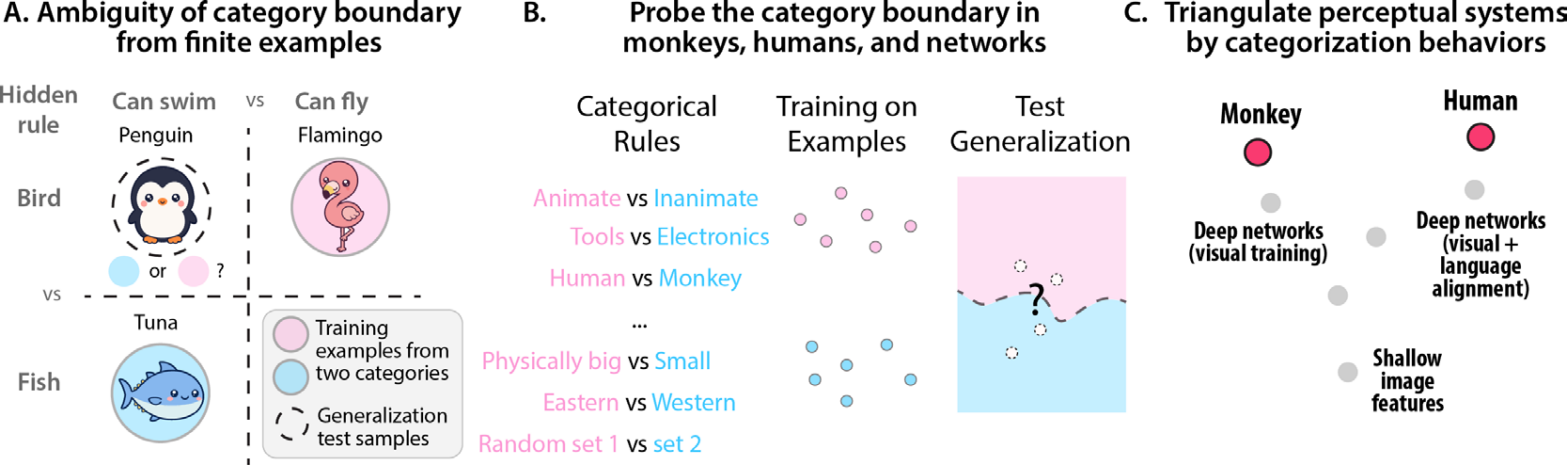
How a learner carves up the visual world, and how the carving can be measured. (**A**) The problem. A learner is rewarded for placing a flamingo in one group and a tuna in another. Two rules explain these examples equally well: bird versus fish (rows), or flies versus swims (columns). The rule is hidden, so the learner must infer it. Where the learner places a new example, such as a penguin, reveals which boundary it inferred, and so reveals something about its built-in assumptions. Dashed lines mark the two candidate boundaries. Pink and blue circles mark training examples from the two categories; the dashed circle marks a new item used to test generalization. (**B**) The paradigm used by Zhang et al. The researchers choose a pair of categories as a hidden rule, then show the monkeys training images drawn from each category with feedback (filled pink and blue dots). New images, never seen before, are then presented to test generalization (dashed open dots). The dashed curve marks the boundary the learner has inferred. Rules ranged from animate versus inanimate to physically large versus small, and Eastern versus Western objects. A control rule assigned images or object types to two arbitrary sets that shared no meaning. (**C**) The comparison. Using categorization behavior across all tasks, the Zhang et al. place monkeys and humans within the same space spanned by computational models. Red circles mark the two species. Grey circles mark models, ranging from shallow image features such as color and texture to deep networks – models with many processing layers trained to label images; unlabeled gray circles are other models. Monkey behavior lay closest to networks trained on images alone, whereas human behavior lay closest to networks trained to align images with language. Redrawn schematically from Figure 6E in Zhang et al., 2026.

This is an important distinction because different learners can achieve similar accuracy yet carve the visual world along different seams. For example, an artificial neural network may classify a cat rendered with elephant skin as an elephant, while a person still sees a cat (Geirhos et al., 2018). Although both systems may perform similarly on standard classification tests, matching accuracy does not necessarily imply matching category boundaries: one system partitions the visual world by texture, while the other one categorizes it by shape. Generalization to unfamiliar examples can therefore reveal not just whether a learner has learned to classify, but what principle it has learned to classify by.

Monkeys have been extensively used to study vision and learning. They have sophisticated primate vision, but do not possess human language nor cultural knowledge. Their brain activity supporting categorization has been mapped in detail and has been shown to be very similar to humans. However, it has so far remained unclear if they possess the ability to classify objects into a range of high-level categories similar to humans (Hung et al., 2005; Fabre-Thorpe, 2003). Now, in eLife, Gouki Okazawa and colleagues at the Chinese Academy of Sciences – including Han Zhang as first author – report new insights into high-level categorization in monkeys (Zhang et al., 2026).

Zhang et al. scaled up a binary decision-making paradigm by training monkeys on samples from two categories and then testing their generalization to new images (Figure 1B). Three monkeys completed more than ten tasks across roughly 315,000 trials, categorizing from animate versus inanimate to fire-related versus water-related objects. The monkeys learned common categories within two to three days and applied them to new images, including object types absent from training. Control tests showed that they were not memorizing pictures or leaning on a single cue such as color or fur texture, suggesting they learned the underlying rule.

Two results stand out. First, when images or object classes were randomly assigned into two groups, the monkeys learned more slowly and generalized poorly, echoing humans and networks trained on randomly assigned labels (Zhang et al., 2017; Shepard et al., 1961). Just as human categories are not arbitrary conventions (Rosch et al., 1976), monkeys too find some carvings of the visual world far easier to learn than others. Second, the failures were as informative as the successes. Monkeys generalized rules with reliable visual structure, such as indoor versus outdoor scenes, or physically large versus small objects (Konkle and Oliva, 2012), but failed to generalize on Eastern versus Western cultural objects – a task well performed by humans. This gap hints at where vision ends and cultural knowledge begins.

Pooling the performance of every task, Zhang et al. triangulated monkeys and humans against a set of artificial networks (Figure 1C). Monkey behavior most resembled networks trained on images alone – without language input, while human behavior was closer to networks trained to align images with language (Radford et al., 2021), highlighting a previously underemphasized distinction between human and monkey visual function.

Albeit simple in design, the sheer scale of the task makes the work of Zhang et al. truly compelling. The resulting behavioral data gives future computational models of primate vision a demanding target. A good model should reproduce not only average accuracy, but also learning speed, error patterns and failures on unfamiliar images.

The study also closes a loop: primate vision inspired modern computer vision; artificial intelligence inspired scalable ways to measure learning and generalization. Zhang et al. now bring those methods back and study primate learning in unprecedented detail.

Open questions remain. Do monkeys truly understand what it means to be alive, or have they simply learned to separate images into groups based on visual patterns? Would monkeys raised with richer experience learn the cultural rules they failed here? And could a model predict, before the experiment, which rules a monkey will fail to learn? These are questions the two fields can now ask together.

## References

[bib1] Fabre-Thorpe M (2003). Visual categorization: accessing abstraction in non-human primates. Philosophical Transactions of the Royal Society of London. Series B, Biological Sciences.

[bib2] Geirhos R, Rubisch P, Michaelis C, Bethge, MA, Wichmann F, Brendel W (2018). ImageNet-Trained CNNs Are Biased towards Texture; Increasing Shape Bias Improves Accuracy and Robustness. arXiv.

[bib3] Goodman N (1955). Fact, Fiction, and Forecast.

[bib4] Hung CP, Kreiman G, Poggio T, DiCarlo JJ (2005). Fast readout of object identity from macaque inferior temporal cortex. Science.

[bib5] Konkle T, Oliva A (2012). A real-world size organization of object responses in occipitotemporal cortex. Neuron.

[bib6] Radford A, Kim JW, Hallacy C, Ramesh A, Goh G, Agarwal S, Sastry G, Askell A, Mishkin P, Clark J, Krueger G, Sutskever I (2021). Learning Transferable Visual Models From Natural Language Supervision. arXiv.

[bib7] Rajalingham R, Issa EB, Bashivan P, Kar K, Schmidt K, DiCarlo JJ (2018). Large-scale, high-resolution comparison of the core visual object recognition behavior of humans, monkeys, and state-of-the-art deep artificial neural networks. The Journal of Neuroscience.

[bib8] Rosch E, Mervis CB, Gray WD, Johnson DM, Boyes-Braem P (1976). Basic objects in natural categories. Cognitive Psychology.

[bib9] Shepard RN, Hovland CI, Jenkins HM (1961). Learning and memorization of classifications. Psychological Monographs.

[bib10] Zhang C, Bengio S, Hardt M, Recht B, Vinyals O (2017). Understanding Deep Learning Requires Rethinking generalizaƟon. arXiv.

[bib11] Zhang H, Zheng Z, Hu J, Wang Q, Xu M, Zhou Z, Li Z, Okazawa G (2026). A battery of image classification challenges reveals shared and distinct object categorization behavior across monkeys, humans, and deep networks. eLife.

